# Evaluation of the designed multi-epitope protein of *Brucella melitensis* in guinea pigs

**DOI:** 10.22038/ijbms.2021.54667.12267

**Published:** 2021-06

**Authors:** Mina Saadat, Masoud Gandomkar, Akram Bahreinipour, Mojgan Bandehpour, Bahram Kazemi, Nariman Mosaffa

**Affiliations:** 1 Department of Medical Biotechnology, School of Advanced Technologies in Medicine, Shahid Beheshti University of Medical Sciences, Tehran, Iran; 2 Cellular and Molecular Biology Research Center, Shahid Beheshti University of Medical Sciences, Tehran, Iran; 3 Veterinary Institution, Tehran, Iran; 4 Department of Immunology, School of Medicine, Shahid Beheshti University of Medical Sciences, Tehran, Iran

**Keywords:** Brucella melitensis, Epitope mapping, MALDI-TOF, Peptides, Vaccine, 2-Dimensional gel - electrophoresis

## Abstract

**Objective(s):**

One of the causes of human and animal zoonotic infections is *Brucella melitensis*, which is transmitted to humans through dairy products. It seems for prevention of human infection we might protect the livestock by an efficient protein as a vaccine candidate. For this purpose, the use of immunogenic proteins of bacteria is able to create immunity the same as the traditional vaccines.

**Materials and Methods:**

In this study, by finding the immunogenic antigens of this bacterium by 2-dimensional gel electrophoresis and MALDI-TOF methods and also the proteins reported in other studies, we found the epitopes of the bacterial antigenic determinants* in silico.* Nineteen peptides of T and B epitopes were selected. They were ligated with linkers and after gene synthesis, the designed polypeptide was expressed in *Escherichia coli* BL21. The purified recombinant MEL protein mixed with chitin was injected subcutaneously into three 300 g male guinea pigs three times. Also, PBS control and Rev.1 commercial vaccine groups were considered.

**Results:**

The results show that MEL polypeptide is equal to the Rev.1 vaccine in stimulating secretion of IFNγ and IL2 and specific IgG. High levels of IL-2 emphasize the activation of the cellular immunity, and in particular comparison of PI in guinea pig’s spleen cells treated with recombinant MEL protein on days 0 and 5 show that it has significant proliferation compared with PBS unstimulated cells.

**Conclusion:**

This recombinant protein could be a subunit protein with sufficient efficiency in stimulating the humoral and cellular-mediated immune system against *B. melitansis.*

## Introduction

Brucellosis is the most common bacterial disease of humans and animals worldwide, with over 500000 infections in humans annually (1, 2). Brucellosis causes high economic loss in livestock by abortion and is known as a life-threatening multisystem disease in humans (3-5). It is an endemic infection in many parts of the world, including the Middle East, Africa, Latin America, Central Asia, and many regions of the Mediterranean basin; and Iran is an endemic region for brucellosis (6, 7). Therefore, this infection is an enormous challenge for health. *Brucella melitensis* is the main cause of brucellosis in sheep, goats, and humans (8). The dairy products of infected animals may contain large numbers of viable organisms (9, 10). The live vaccine B. melitensis strain Rev.1 is used worldwide for prevention of brucellosis in small ruminants (11, 12). The potential risks to veterinarians have always been raised, and confirmation of Rev.1 injection in humans with high-dose experimental inoculation has been demonstrated in volunteers (13). Therefore, a subunit vaccine that is protective against *B. melitensis* is necessary. A number of studies with computational approaches have predicted epitopes of antigens that are effective in stimulating the immune system and have used these findings in an experimental study aimed at obtaining an epitope-based vaccine (14-17). An immunogenic multitope protein containing antigenic epitopes from several dominant parts of the bacterial structure may provide protective immunity against brucellosis (18). Establishing an effective immune response against Brucella infection requires cell-mediated responses, particularly Th1, which is associated with the production of interferon-gamma, and humoral response, which produces specific antibodies (19-21). In this study, we employed a reverse vaccinology approach and bioinformatics analysis to find protective complex candidates for induction of both cellular and humoral immunity in sheep and goat brucellosis.

## Materials and Methods

***Antigenic protein identification***

*Choice of anti-sera and bacterial strain*

To analyze the *B. melitensis *immunoreactive protein profile, a total of 10 sheep sera samples naturally infected with *B. melitensis *(contains antibody) and ten non-infected, and seven *Mycobacterium* infected sheep (as the negative controls) sera were collected from the Meysam slaughterhouse (Tehran, Iran) for this study. The characterization of negative or positive status of serum samples was performed by using the confirmation serological methods including, Rosebengal, Tube wright, and Coombs wright (22). We created a serum pool of each group of samples. The bacterial strain for *B. melitensis* used in this study was taken from the culture collection of the Razi vaccine and serum research institute (Karaj, Iran). 

*Extraction of structural proteins*

To isolate the whole-cell structural proteins, the strain was cultured for 48 hr in Tryptic Soy Broth (TSB) at 37 °C with shaking (23, 24) at the Razi Vaccine and Serum Research Institute (Karaj, Iran). Briefly, the culture was harvested by centrifugation and washed twice with phosphate buffer saline. The cells were reconstituted in 80% ethanol and centrifuged. Subsequently, the cell pellet was suspended in lysis buffer (7 M urea, 2 M thiourea, 4 % (w/v) CHAPS, 40 mM Tris base, and 0.002% Bromophenol Blue), sonicated on ice for 1 min (duty cycle: 1.5, amplitude: 90%, UP100H), centrifuged at 12,000g for 10 min at 4 °C and the supernatant was collected and kept at -20 °C.

*Two-dimensional polyacrylamide gel electrophoresis (2DE) and Western blotting*

Seven-centimeter immobilized pH gradient (IPG) strips with nonlinear range of pH 3-10 (Bio-Rad) were used to perform isoelectric focusing (IEF) and processed as described previously (25). For the Immune blotting procedure, 25 μl of total protein lysate was separated using a precast preparative 15% SDS–PAGE and blotted onto a nitrocellulose membrane (Sigma–Aldrich) by semi-dry transfer cell (Bio-Rad, Hercules, CA, USA) and processed as described previously. We used infected sheep sera (1:500 diluted) as the primary antibody source and the goat anti-sheep total IgG HRP-conjugated as a secondary antibody for *B. melitensis* protein antigens detection. The visualization of signals was performed with substrate buffer (Diaminobanzoic acid), 10 mL PBS, and 30 μl hydrogen peroxidase.

*Protein identification by mass spectroscopy and mascot analysis*

The protein bands which were reacted with infected sera immunoglobulin were excised from the SDS–PAGE gels and destained, and protein extraction was proceeded by the manufacture process described previously (25). Then, the isolated peptides were purified using 25 μl of acetonitrile, mixed, and dried using a vacuum centrifuge. The protein sample preparation was proceeded according to standard techniques. Subsequently, a database exploration for protein characterization was carried out using MS/MS ion search (MASCOT, www.matrixscience.com) against all entries of NCBInr. Protein identification is valid when more than 2 peptides match and MOWSE scores are significant (*P*<0.05).

***Construction design and immuneinformatics***

*Amino acid sequence retrieval, epitope predicting, and plasmid designing*

Identified proteins with significant MOWSE scores were considered for the antigens of *B. melitensis*. To assess the primary protein structure of each antigen the ProtParam tool was used to obtain the molecular weight, isoelectric point (PI), grand average of hydropathicity (GRAVY), amino acid composition, and other physicochemical features. The T cell epitopes from all of the obtained antigens (considering MHC I&II) were predicted, using the online prediction server Immune Epitope Database (IEDB) (https://www.iedb.org/). Online servers like ABCpred (https://www.bio.tools/) were applied to predict the linear B-cell epitopes**. **The IEDB server predicts the peptides based on Chou and Fasman beta-turn, Karplus and Schulz flexibility, Emini surface accessibility, Kolaskar and Tongaonkar antigenicity, and Parker hydrophilicity. The selected epitopes are fused using glycine-serin amino acid-rich linkers in such a way that the best poly-epitope antigen (named MEL) is obtained, which expresses all of the epitopes at the surface. For computation of several physicochemical parameters of the MEL protein, we used ProtParam at (http://expasy.org/tools/protparam.html). After validation of the construct’s confidants, the poly-tope protein coding sequence was synthesized chemically by GeneCreate Biological Engineering Co (China).

*In silico evaluation of multi-epitope protein*

The VaxiJen v.2.0 server at (http://www.jenner.ac.uk/VaxiJen) was used for prediction of the immunogenicity of the MEL protein. The accuracy of this server based on the type of study varies from 70% to 89%. The prediction of protein allergenicity was performed via the AlgPred webserver at (http://www.imtech.res.in/raghava/algpred/). The accuracy of the hybrid prediction approach is near 85% with a threshold of 0.4. I-Tasser generates reliable protein models without close homologs in the Protein Data Bank (PDB). *P*-value and RMSD indicate the relative global quality and absolute local model quality, respectively. 

***Cloning, expression, purification, and confirmation***

The computational approved sequence was synthesized into the *pET22b *expression vector (GeneCust, Luxembourg S.A) using restriction enzymes (*SacI *& *HindIII*) and named *pETmel22b*. The transformed BL21 (DE3) (Invitrogen, Germany) strain of *E. coli *was considered for protein expression*. *Protein concentration was measured using the Bradford assay. Then, the bacterial lysate was electrophoresed onto a 12% Sodium dodecyl sulfate-polyacrylamide gel (SDS-PAGE) beside the protein marker (Fermentas, Lithuania). The expression of recombinant protein was compared with that of the control samples’ *E. coli *BL21without plasmid. The recombinant multi-epitope protein was purified under native conditions on His-Tag resin (Invitrogen, Germany) according to the manufacturer’s guidelines. The purified MEL protein and the controls were separated by the SDS-PAGE method using 12% and transferred onto a nitrocellulose membrane (Sigma-Aldrich, USA) by blotting BioRad system. To assay, the antigenicity of the purified multi-epitope peptide, the membrane, after blocking with a solution containing 3% skim milk (Fluca), was incubated for 100 min at 25 °C with the sheep’s 1:200 dilutions of sera, obtained from 20 sheep in Razi Institute, which was previously confirmed by a serology test. The membrane was washed and incubated with goat anti-sheep IgG Alkaline phosphatase conjugate (1:5000) (Abcam, UK) as the secondary antibody for two hours at 37 °C. Following the washing stage, the immune complex was visualized by incubating the membrane with a solution containing NBT-BCIP for 15 min at 37 °C. The reaction was stopped using distilled water.

***Preparation of chitin microparticles as an adjuvant in immunogenic complex***

The commercial powder of the chitin microparticles was suspended in distilled water, sonicated, and filtered using a 40-µm filter (BD Falcon, Mexico) (26). The pellet of the microparticles after centrifugation (2800g for 10 min) was oven-dried at 50 °C. Particle size and distribution were analyzed using a laser particle size analyzer. Before applying it as an adjuvant in the immunogenic complex, we checked it for the presence of LPS using the Limulus Amebocyte Lysate kit (Cambrex, USA). 

***In vivo evaluation of multi-epitope protein immunogenicity***

*Animals*

We performed the study on 9 guinea pigs (*Cavia porcellus) *weighing from 300 g in the majority of the experiments, obtained from the Medzist company, and kept under isolation as a routine. They were graded into 3 groups called PBS group, Rev.1 vaccine, and MEL protein. We have followed all ethical principles of research on laboratory animals under the National Institutes of Health guide for the Care and use of Laboratory Animals (NIH Publications No. 8023, revised 1978) (Ethics Code: IR.SBMU.REC.1396.126). 

*Immunization of guinea pigs*

The animals were immunized by injection subcutaneously. The injection solution was 200 µl containing 10 mg MEL protein and chitin microparticles (100 mg/mL) for the MEL group; 200 µl PBS and 200 µl Rev.1 vaccine were used for the two other groups.

*Samples collection and primary cell culture*

Before sacrification of the animals, the blood of each experimental group was collected from the cardiac puncture to perform the antibody ELISA (27, 28) (Enzyme-Linked Immune Sorbent Assay) and also for bactericidal antibody responses test. After sacrifice and cutting off guinea pigs’ lower jaws the spleens were dissected, the lymphocytes were isolated by Ficol 70(Sigma), and then cultured in RPMI (Bio sera) (with penicillin 100 u/ml and streptomycin 100 µg/ml) at 37 °C, 5% Co_2_, and 80% humidity at 6x10^6^ density of cells for proliferation assay. Proliferation stimulation by adding injected components was performed 24 hr after cell culture. Liver tissues were kept in formalin until slide staining for examination of any pathological changes of the protein.

*Measurement of specific IgG against MEL protein in guinea pig’s sera by ELISA*

For evaluation of IgG production stimulation in guinea pig’s sera, after an immunogenic complex injection, different concentrations of purified protein in 1x PBS were coated on a crystal-grade polystyrene 96-well microtiter plate (SPL Life Sciences) (29). The volume of 100 μl of 1/200 diluted serum samples in 1x PBS was added to each well. The plate was left at room temperature for 2 hr . The plate was washed three times, and 100 μl of HRP-conjugated rabbit anti-guinea pig IgG (1:10000 dilutions in 1x PBS) was added to each well. After 1 hr and re-washing the plate, 100 μl of 3,3’,5,5’-Tetramethylbenzidine (TMB) substrate was added and after 15 min at room temperature, the reaction was stopped by 50 μl of 2N H_2_SO_4_. The Optical Density (OD) of each well was assayed by ELISA reader at wavelength 450 nm with reference wavelength 630 nm. The method was applied for all of the serum samples in 1:200 dilutions and with 1 μg/ml of purified protein. All of the 25 anti-*Brucella* IgG negative samples were considered to set a cutoff value using mean ± 2SD. 

*Measurement of IL2, IL5, IL4, IL10, TNFα, and IFNγ cytokine genes expression by real-time q-PCR*

Total RNA was extracted from spleens by using a Gene All Kit(Korea) according to the manufacturer’s guidelines. RNA was then treated with 5 U of DNase I (Fermentas, Lithuania). RNAs were measured using a Biophotometer (Eppendorf, Germany). To synthesize cDNA, 2 μl of oligo (dT) primer, 2 μl of 2.5 mM stock of dNTPs (Invitrogen), and 16 μl of RNA were incubated at 65 °C for 5 min and then put on ice for 3 min. The following reagents were added: 4 μl of 5× buffer (Fermentas) and 0.5 μl of 200 unit/μl Revert Aid^TM^ (Fermentas) followed by keeping at 42 °C for 50 min. **Table 1** lists the six guinea pig cytokines and Gapdh as housekeeping genes (GenBank). 

Real-time RT-PCR was carried out with a Bioneer Master mix (Korea) using 1 μl of the cDNA in a volume of 20 μl with the specific primers mix. Quantitative PCR was carried out in the LightCycler 480 for 45 cycles using the following program: denaturation at 95 °C for 10 sec, annealing at 60 °C for 10 sec, and extension at 72 °C for 15 sec. Melting curve analysis was achieved after the amplification phase. Cytokine amplification was confirmed by 2% agarose gel electrophoresis.

*Lymphocytes proliferation assay by flow cytometer*

The proliferation character of the three groups was carried out by adding the MEL, PBS, and Rev.1 vaccines separately 24 hr after cell culture. Following 6 day incubation of cells at 37 °C in a 5% CO_2_ humidified incubator, they were harvested and the proliferation rate was assessed via measuring fluorescent label carboxyfluorescein diacetate succinimidyl ester (CFSE) (Life Technologies, USA) uptake. The assay described in this section is used to track proliferating cells due to the CFSE intracellular fluorescent label (30). A FACS Aria II flow cytometer (BD Biosciences, San Diego, CA, USA) was used for the flow cytometry technique, Which has two power lasers: 488 nm and 635 nm. The data were analyzed with FlowJo software (TreeStar, Ashland, OR, USA).

***Protein toxicity assay on guinea pigs’ liver tissue by microscopic evaluation***

The histology of liver samples of guinea pigs was prepared with paraffin and cut. After Hematoxylin and Eosin staining, they were examined by a specialist.

***Statistical analysis***

All analyses were performed in three versions. Values were assumed as means±SD. Statistical analysis was performed by one-way analysis of variance (ANOVA) and then multiple comparison tests in Turkey’s. *P*-values less than 0.05 were considered to be statistically significant. REST^@^ 2009 Software (Qiagen, Hilden, Germany) was used to analyze RT-qPCR data. 

## Results

***Immunoreactive proteins in Brucella melitensis cells***

**Figure 1:**
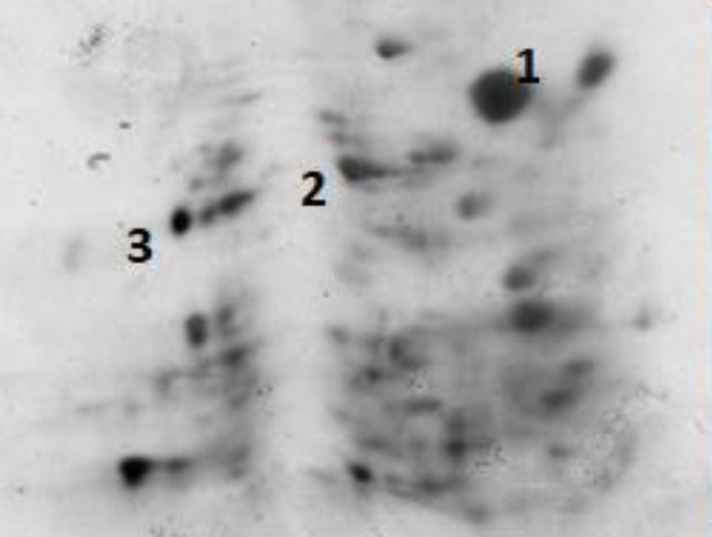
2D gel electrophoresis of *Brucella melitensis* lysate. The spots number 1, 2, and 3 were positive in interaction with infected sheep pooled serum

Western blotting of bacterial strains with confirmed naturally infected animal sera showed that there are several antigenic proteins in *B. melitensis* cells that induce the humoral immune system in sheep. Figure 1 demonstrates the analysis of *B. melitensis* proteome by 2DE and we selected 3 sharp spots that were positive in interaction with infected sheep pooled serum for MALDI TOF MS spectroscopy. 

***Proteomics analysis of B. melitensis***

The protein bands which reacted with infected sera were prepared for analysis by MALDI TOF MS/MS. Protein identification is valid when more than 2 peptides match and MOWSE scores are significant (*P*<0.05) (Table 2). Table 3 shows isolated proteins of *B. melitensis* that referred to from other articles.

***Selected epitopes for construct design***

Using servers that were described, the highest scored T-cell and B-cell epitopes for *B. melitensis *immunogen proteins were selected and sorted in Table 4. 

***Predicted structure and physicochemical properties***

**Figure 2:**
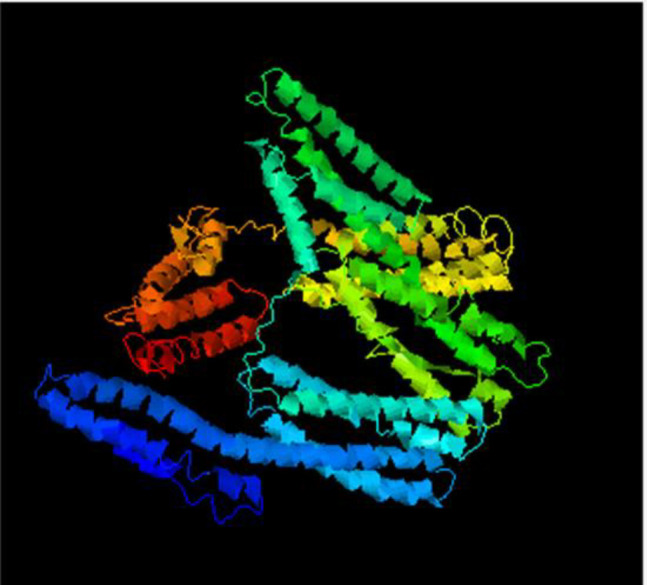
Global model of MEL protein (6bfiA) by I-TASSER

The 3D structure of the MEL protein was predicted by the I-TASSER server (C516795 project) (Figure 2). The best template resulting for the homology modeling was **6bfiA **with a normalized Z-score 1.35 which means a good alignment and shows the high quality of the model. The tertiary model quality score and limited errors were analyzed using the pGenTHREADER server, which predicted the C-score to be 1.13, and computed TM-Score 0.57±0.14 and accounted RMSD 10.6±4.6 which represents a model of correct global topology. 

ProtParam server administered basic physicochemical parameters of the protein. The number of amino acids was considered 431 with a molecular weight of 45116.47 Da. The pI value was 6.30, and the computed half-life of the MEL protein was 30 hr (mammalian reticulocytes**, ***in vitro*), >20 hr (yeast**, ***in vivo*), and >10 hr (*Escherichia coli* *in vivo*). The instability index was computed to be 38.29 which classified it as a stable protein. The GRAVY index score was 0.161 which shows the hydrophobic nature of MEL by Kyte-Doolittle and Hopp Woods formula. VaxiJen overall prediction of the protective antigen for MEL represented that it is a probable antigen with a score of .0.5772. 

***The MEL recombinant protein production and confirmation by Western blotting***

The MEL fragment was cloned into the pET22b expression vector (Figure 2) and then expressed in *E. coli* and purified. Western blotting analysis of MEL recombinant protein revealed with infected sheep serum and His Tag monoclonal antibody showed that the protein has expressed after induction and purified successfully.

***Specific IgG detection in guinea pigs’ sera***

**Figure 3:**
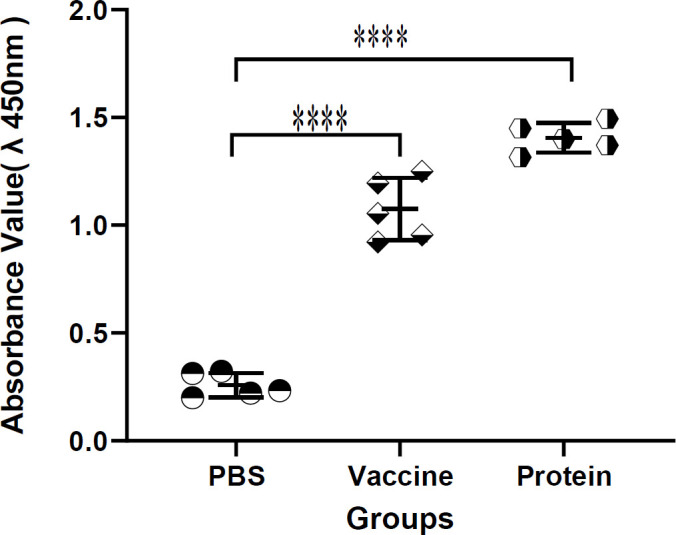
Rate of absorption in the sera of guinea pigs treated with MEL, Rev.1 vaccine and PBS

To determine whether recombinant *B. melitensis* MEL protein elicited antibody responses in the guinea pig, we obtained sera from five guinea pigs experimentally injected with recombinant *B. melitensis* MEL protein, sera from five guinea pigs injected with the whole *B. melitensis* antigen (Rev.1 vaccine), and sera from five guinea pigs injected with PBS as the negative control. The levels of IgG specific for recombinant MEL protein and Rev1 vaccine with a mean A450 of 1.405± 0.068 and 1.075± 0.144, respectively, did not differ between guinea pigs. In contrast, for two groups of animals infected with *B. melitensis* antigen, the levels of IgG-specific increased significantly above those measured in guinea pigs infected with PBS with a mean A450 of 0.258±0.056 (*P*<0.0001). These results suggest that recombinant MEL protein is capable of responding to guinea pigs like the *B. melitensis* Rev1 vaccine (Figure 3).

***MEL protein and sheep serum IgG antibody complex-forming***

**Figure 4:**
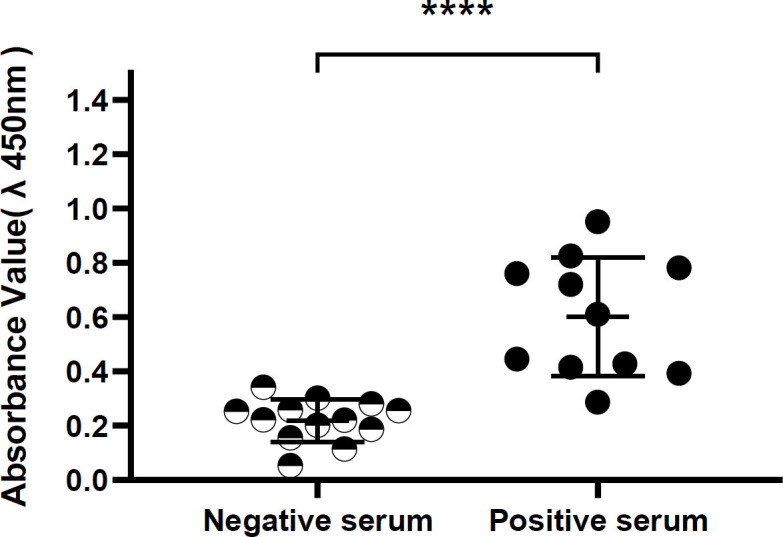
Absorption of MEL protein in healthy sheep sera and sheep with *Brucella melitensis*

Thirteen positive and twelve negative serum samples from sheep for antibodies to the whole *B. melitensis* antigen were examined by ELISA for existence of specific antibody (IgG) for recombinant *B. melitensis* protein. The results are shown in Figure 4 that levels of IgG against the recombinant protein (MEL) were significantly higher in positive sera with a mean A450 of 1.375± 0.145 compared with negative sera with a mean A450 of 0.328± 0.120 (*P*<0.0001) (Figure 4).

***Cytokine genes expression evaluation by real-time RT PCR***

**Figure 5:**
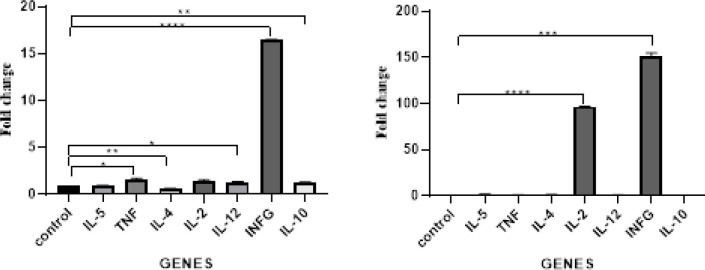
Expression levels of Cytokines. Cytokine RNA transcriptions were compared between MEL protein (right), Rev.1 conventional vaccine (left), and PBS control groups

As shown in Figure 5, Real-time RT-PCR analysis of immunized guinea pigs’ spleen lymphocytes in 3 groups showed that the expression levels of IL-2 and INFγ increased in guinea pigs immunized with MEL protein significantly. While a decrease in TNFα, IL-4, IL-12, IL-10, and IL-5 was observed in this group compared with the controls (Rev.1 and PBS). 

***Lymphocyte proliferation assay***

To assess the lymphocyte proliferation activity, the CFSE-labeled cells were cultured with purified protein following five days of culture, the proliferation rate of the cells was assessed via measuring CFSE uptake.

**Figure 6:**
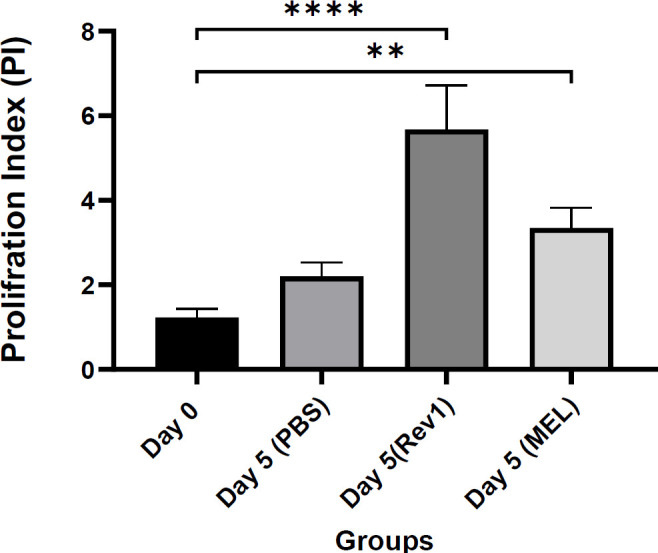
Analysis of the splenocytes proliferation rate (%) after treatment with antigen (Flowjo V7.6.1). Cells in culture with MEL exhibited significant proliferation after 5 days compared with the PBS group. The difference was also significant in the MEL group compared with the PBS group (*P*<0.001). Significance was flagged with star(s); ** *P*≤0.01 and **** *P*≤0.0001

Splenocyte proliferation was assessed by tracking the decrease in CFSE fluorescence in proliferating cells (CD3-gated). The percentage of the proliferation rate was compared between the groups as demonstrated in Figure 6. The results showed that the cells cultured with the MEL antigen group exhibited significant proliferation compared with PBS unstimulated cells.

***Microscopically evaluation***

Histopathological evaluation revealed that in all cases of experimented and sham groups mild portal and/or parenchymal hepatitis (mononuclear and polymorph nuclear cells), vacuolar degeneration, cell swelling, and individual necrotic cells were seen that were not related to the treatment (Figure7). 

**Table 1:** Primers used for cytokine gene expression assay by real-time qPCR

Gene name	Forward primer (5’-3’)	Reverse primer (5’-3’)
IL4	tgacggtcattctcttctgcctc	aggagagtgtgttgaggtgctg
IL-2	gttatgctttctcagagcaaccc	gctaaatttagcacctgctccac
IL12	acctccctagggcctcaccag	ggccttgtgaagttcactgttc
IL5	ggaagctctggcaacactattc	tgcttcactctccgtcgctcc
IL10	gccagccaaggcacgaacacc	accctgccaaaggcagctcgg
TNFα	tgcactttggggtgatcggcc	agccaccggcttgtcattatcg
IFNγ	atgttggctcttcagttctg	catctgagttatctgcattc
Gapdh	cgagacaagatggtgaaggtc	cattgatggctacaatatccact

**Table 2:** *Brucella melitensis* identified proteins by MALDI TOF MS/MS

**number**	** Protein **	**MW (kD)**	**Location**
1	ABC transporter ATP-binding Protein	100	Bacterial pellet
2	Chaperonin 60	70	Media
3	Aldehyde Dehydrogenase	40	Media

**Table 3:** *Brucella melitensis* identified proteins by other studies (2DE)

**Reference**	**Selected proteins**
	25 kDa outer membrane protein-omp25
	BvrR (*Brucella* virulence-related Regulatory protein) BvrS (*Brucella* virulence-related Sensory protein)
	BtpA&BtpB (*Brucella* TIR domain-containing protein A&B), TcpB (‎TIR domain-containing protein from *Brucella)*
	TIRAP9 (‎TIR domain-containing adaptor protein)
	p-type DNA transfer protein VirB5
	Type IV secretion system protein virB10
	Type IV secretion system protein VirB11
	Type IV secretion system protein virB3
	D-erythritol 1-phosphate dehydrogenase
	Erythritol kinase
	D-erythrulose 1-phosphate 3-epimerase
	L-erythrulose 1-phosphate isomerase& D-erythrulose 4-phosphate isomerase

**Table 4:** Epitope mapping outcome of *Brucella melitensis* immunogen proteins

T-cell MHCI binding Epitopes*	B-cell Epitopes*
VPAPVEVAPQYSWAG	KSLVIVSAALLPFSATA
EASATQTI ALVDDDRNILTSVSIALESEGY	EPVTLTVTEFLILHSLAQRPGVVKS
ERRARRQRSVFLRRYLSPLRKFLGQY	ERMAVFRVFGVVSAVMVILSLFLASTIANPLR
KQSLSSMRTTASATMEAEEEYDFFISHASEDKEAFVQDLV	SEDKEAFVQDLVAALRDLGAKIFYDAYTLKVGDSLR
ELAELTRLPTIFAYEAACKLDP	RRGQVRIEYEFIPVQPFLTVADFD
K MSELERATRDGAAIGKKRAD	SEDKEAFVQDLVAALRDLGAKIFYDAYTLKVGDSL
AHKAQQAISSAKSLSTQKSKMSELERATRDGAAIGKK	SEDKEAFVQDLVAALRDLGAKIFYDAYTLKVGDSL
ALTVTSTAHAQLPVTDAGSI	SEDKEAFVQDLVAALRDLGAKIFYDAYTLKVGDSL
MTQENIPVQPGTLDGERGLP	GRTRKVLLFLFVVGFIVVLLLLLVFHMRG
MMSNRSDFIVPDEAAVKRAA	LPYEIIRRLLYLVVDVVVHVHNGVHDG
MTTAPQESNARSAGYRG	RPAMLFGVPVIPLVIVGGSIVLLSVWISMFILPIVPIVLVMRQI

* These are the highest scored T-cell and B-cell epitopes

## Discussion

Iran is one of the endemic regions for brucellosis with a noticeable incidence of brucellosis among human and domestic animal populations.  According to the annual report of the Iranian Centers for Disease Control, the most important causes of human brucellosis are *B. melitensis* and *B. abortus *(43, 44). *B. melitensis* Rev.1, an attenuated smooth strain was able to control *B. melitensis* infection and is currently used as the only vaccine for the prophylaxis of caprine brucellosis (45-47). However, major problems like the ability of this strain to cause brucellosis in humans (48) and creation of resistance to streptomycin used to treat brucellosis, have prevented its use for human vaccination (49). Therefore, a subunit vaccine that is protective against *B. melitensis* is in demand. Many studies similar to our research have reported that a multitope vaccine containing protective epitopes from several immunodominant proteins may exert protective immunity against brucellosis (50). Thus, we employed a reverse vaccinology approach to identify immune-reactive proteins of bacteria and access a potential vaccine candidate for goat brucellosis. Bacterial cell structural and secreted antigens could be the most critical selections for designing vaccines since they might participate in the initial interaction with the host cells. In the production of good and ideal vaccines against brucellosis important functions of the immune system must be strengthened and activated.

Each of the selected peptides from the proteins obtained from this study and the results of previous research are able to stimulate the immune system as peptide vaccines. By connecting them and making a recombinant protein (MEL) and forming a proper folding, we were able to stimulate the guinea pig’s immune system even more effectively than the commercial *Brucella* vaccine, which is a weakened bacterium and can return to the active form. One of the properties of small phagocytosable chitin particles is that they activate alveolar macrophages, leading to the expression of cytokines such as IL-12, tumor necrosis factor-α (TNFα) and IL-18, and then NK cells producing INF-γ (51). Therefore, the use of chitin as a safe adjuvant has contributed to the immunogenicity of the recombinant MEL protein. There has been much evidence demonstrating that the gamma interferon (IFN- γ)-mediated T helper 1 (Th1) immune response is vital and important for the control of *Brucella *infection (19, 52-54). After ruminant vaccination, IFNγ production by Th1 is reported (55). The results from this study indicated that approximately high expression levels of Th1 cytokines (IFN-γ and IL-2) were observed in guinea pigs immunized with Rev-1 vaccine and MEL recombinant protein. High levels of IL-2 emphasize the activation of cellular immunity and in particular, Th1 lymphocytes which are aimed at the elimination of pathogens. In this study guinea pigs immunized with MEL protein or Rev.1 vaccine produced significantly higher specific IgG levels compared with the PBS group. The profile of the antibody response is a reaction to the T helper cell type, all of these results indicate the induced Th1 response against MEL protein. A comparison of PI in guinea pig spleen cells treated with recombinant protein (MEL) on days 0 and 5 suggested that MEL protein has significant proliferation compared with PBS unstimulated cells. 

Our results suggest that this recombinant protein could be a subunit protein together with chitin with sufficient efficiency in stimulating the humoral and cellular-mediated immune system against *B.* *melitansis* compared with common live attenuated   *B. melitensis* Rev.1 vaccines. 

## Conclusion

The MEL recombinant protein containing determinant peptides of *B. melitensis* with chitin could be a potential subunit immunogenic complex against *B. melitensis *with magnificent ability in inducing both types of humoral and cellular-mediated immunity without any toxicity compared with common live attenuated *B. melitensis *Rev.1 vaccine.
